# Developmental Indicators of *Chrysomya nigripes* Aubertin under Different Constant Temperature Conditions and an Application Case for Estimating the PMI_min_

**DOI:** 10.3390/insects14090729

**Published:** 2023-08-25

**Authors:** Yi Guo, Gengwang Hu, Liangliang Li, Mingqing Liao, Jiangfeng Wang, Yu Wang, Luyang Tao

**Affiliations:** 1 Department of Forensic Medicine, Soochow University, Suzhou 215000, China; 20214221021@stu.suda.edu.cn (Y.G.); 20214221010@stu.suda.edu.cn (G.H.); 20214021012@stu.suda.edu.cn (L.L.); jfwang@suda.edu.cn (J.W.); taoluyang@suda.edu.cn (L.T.); 2 Criminal Police Branch, Zhongshan Public Security Bureau, Zhongshan 528400, China; zsfylmq@163.com

**Keywords:** forensic entomology, *Chrysomya nigripes*, development, postmortem interval, highly decomposed

## Abstract

**Simple Summary:**

As ectotherms, the growth and development of sarcosaprophagous insects at different temperatures can be useful to estimate the minimum postmortem interval. Blow flies are the most common indicator insects for estimating the minimum postmortem interval. *Chrysomya nigripes* Aubertin, 1932, in the family Calliphoridae, shows later colonization and longer durations on carcasses than those of other species in this family. This species can be used to estimate the minimum postmortem interval over shorter periods and can be applied to highly decomposed bodies and those in the skeletonized stage. In the present study, we characterized the development of *C. nigripes* at eight constant temperatures between 16 °C and 37 °C and constructed various models to describe patterns of development. *Chrysomya nigripes* completed the entire developmental process at 19–37 °C. The overall duration of development (±SD) decreased steadily as the temperature increased from 644.9 ± 36.8 h at 19 °C to 191.5 ± 3.8 h at 34 °C and then remained stable with values of 191.8 ± 2.0 h at 37 °C. This study provides comprehensive developmental data for estimating the minimum postmortem interval using *C. nigripes*.

**Abstract:**

*Chrysomya nigripes* Aubertin, 1932, is a Calliphoridae species that colonize the carcass after the bloat phase and remains for long periods. Some early sarcosaprophagous insects complete one generation of development and are no longer associated with the corpse and surrounding environment, while *C. nigripes* larvae and pupae remain, providing a basis for the estimation of the minimum postmortem interval (PMI_min_) for highly decomposed or skeletonized carcasses. However, data on the growth and development of this species are not yet complete. As a result, we studied the developmental patterns of *C. nigripes* at eight constant temperatures ranging from 16–37 °C and constructed various developmental models, including the isomorphen diagram, isomegalen diagram, linear thermal summation model, nonlinear thermodynamic Optim SSI model, and logistic regression model. *Chrysomya nigripes* could not complete the entire developmental process at 16 °C, although it could be completed at other temperatures. The mean developmental times (±SD) of *C. nigripes* from egg to adult at 19 °C, 22 °C, 25 °C, 28 °C, 31 °C, 34 °C, and 37 °C were 644.9 ± 36.8 h, 422.9 ± 20.1 h, 323.1 ± 13.9 h, 246.6 ± 11.2 h, 202.5 ± 1.8 h, 191.5 ± 3.8 h, and 191.8 ± 2.0 h, respectively. The thermal summation constant (K) and lower critical thermal threshold (T_L_) derived from the linear thermal summation models were 4083.00 ± 293.39 degree hours and 12.52 ± 0.83 °C, respectively. In addition, T_L_, intrinsic optimum temperature (T_Φ_), and upper critical thermal threshold (T_H_) estimated by the optimized nonlinear thermodynamic Optim SSI model were 15.76 °C, 24.88 °C, and 38.15 °C, respectively. This study provides more comprehensive developmental data of *C. nigripes* for PMI_min_ estimation.

## 1. Introduction

Although numerous creatures, ranging from microorganisms to saprophagous mammals, are involved in the decomposition process of corpses, insects remain the most prominent and forensically significant of these elements [1]. Researchers believe that forensic entomology is the most effective method of estimating the postmortem interval (PMI) due to several characteristics of insects, such as the wide variety of species, wide taxonomic range, high flying ability, arrival at the body within minutes, regular growth and development, and the presence of distinct succession waves on the corpse [2,3,4]. Previously, forensic pathology assessed PMI, primarily using algor mortis, rigor mortis, livor mortis, corneal opacity, and stomach contents, but these approaches were only effective for short-term cadavers (1–3 days) [5]. Therefore, forensic entomology has unique advantages over traditional forensic pathology methods. These benefits include the ability of forensic entomology to estimate PMI utilizing insects on the corpse for shorter periods but also for highly decomposed bodies and those in the skeletonized stage [6], thereby expanding the estimation window of PMI to a broader time range; this is supported by many worldwide success cases [7,8,9,10].

The time period between when the insect colonizes the corpse and when the corpse is found is usually defined to be the time of colonization (TOC), which is equivalent to PMI if the insect colonizes the corpse immediately after death. However, when using insect evidence to estimate TOC, many possible assumptions must be considered for its interpretation as a PMI estimate. For example, weather station temperatures are representative of death scene temperatures, colonization occurs only during daylight hours, the insects sampled are the primary to colonize, there are no toxins in the decedent, and published developmental data accurately reflect the development of collected specimens [11]. Generally, the developmental patterns of insects are used to estimate the age of insect specimens; however, for the reasons mentioned above, the true PMI is generally underestimated. Accordingly, the estimated developmental duration of insects is usually roughly representative of the minimum postmortem interval (PMI_min_).

Blow flies and flesh flies are the most commonly used indicator insects for estimating PMI_min_ based on their growth and developmental patterns [12,13,14]. However, most of them appear on the corpse earlier, are the first insects to colonize the corpse, and have a relatively short life history; they can generally only be used to effectively estimate PMI within a month [15]. Therefore, identifying indicator insects that arrive later and remain on the carcass longer is crucial for a wider range of PMI estimates. 

*Chrysomya nigripes* Aubertin, 1932 is a species of the Calliphoridae family and is currently considered a forensically important species that has been reported in many succession experiments and cases [16,17,18]. However, unlike other species of this family, it appears on the corpse later, during the bloat phase or advanced decomposition stage, and remains on the corpse for a longer period [19]. Therefore, it is of great value for PMI_min_ estimation after longer periods. This species is mainly distributed in Southeast Asia, specifically in the Philippines, Thailand, Singapore, and Bangladesh. It is also prevalent in southern cities of China [20]. Therefore, it is characterized by heat resistance, not cold resistance, and can still grow and develop in some high-temperature environments, as an indicator insect of PMI_min_ estimation. The growth and development of this species have been studied by only a few researchers, namely O’Flynn [21] and Li et al. [22]. O’Flynn [21] only evaluated a limited developmental duration for *C. nigripes* at 28 °C, while Li et al. [22] provided data on body length and developmental duration for *C. nigripes* at four temperatures. We discovered that *C. nigripes* frequently appeared and was consistently utilized as an indicator insect to estimate PMI_min_ in multiple cases. However, the available data on the developmental pattern of *C. nigripes* were incomplete. Therefore, we collected *C. nigripes* in a field succession experiment and studied their developmental duration, thermal summation, and larval body length in the laboratory at eight constant temperatures ranging from 16–37 °C and established various developmental models at temperatures from 19–37 °C. These models not only provide the lower and upper critical thermal threshold (T_L_, T_H_) and intrinsic optimum temperature (T_Φ_) for the species, but also enable quick calculations of the developmental duration from known temperatures and larval body length or developmental events in real forensic cases to estimate the PMI_min_. This research provides more basic data for estimating PMI_min_ using *C. nigripes*.

## 2. Materials and Methods

### 2.1. Insect Source

A pig carcass was placed in an open space near the Nanlang Public Security Bureau (22°30′ N, 113°23′ E) in Zhongshan City, Guangdong Province, China, in the summer of 2021. Larvae with black bands on their backs on the decaying carcass were found, preliminarily identified as wandering third instar larvae of *C. nigripes*, which were collected and transported to the forensic entomology laboratory of Soochow University for breeding for the establishment of laboratory population.

### 2.2. Laboratory Population Establishment 

The wandering larvae were placed in a 22 cm × 15 cm × 8 cm insect rearing box, which had a 3 × 5 cm ventilated nylon mesh window in the center of the lid, and the box was contained with 2 cm of vermiculite at the bottom. The rearing box was placed in an intelligent light incubator KXG-300 (Yingmin Co. Ltd., Changzhou, China) at 25 °C until pupariation. After pupariation, the box was placed in a 50 × 50 × 50 cm insect-rearing cage, and the lid was removed to allow the newly emerged adults to fly. The rearing cage was set up in a room with a temperature of 28 °C, a humidity of 70% and natural light, and a 12 cm diameter Petri dish filled with a 1:1 mixture of sugar and milk powder. A small plastic box lined with 10 × 6 × 2 cm nanosponges was also contained with fresh water and placed in the rearing cage to ensure nutrient and water intake. In our field succession experiment, we discovered that *C. nigripes* appear after the bloat phase of the carcass and prefer to lay their eggs on the skin of the pig, while most other species arrive at the carcass during the fresh period and lay their eggs in the natural opening of the carcass. Thus, we placed fresh pork in an intelligent light incubator at 31 °C for 12 h and left it to decompose. Following the maturation of the adults, 40 g of decomposed pork was placed in a 50 mL beaker covered with a layer of pig skin, and the beaker was placed in a rearing cage to attract eggs laying. The eggs were observed every hour, and after laying, the eggs were transferred to an intelligent light incubator. This procedure was repeated until the population grew to include around 2500 adults in each rearing cage.

### 2.3. Species Identification 

#### 2.3.1. Morphological Identification

Adult *C. nigripes* were morphologically identified using a Carl Zeiss stereomicroscope (Carl Zeiss, Göttingen, Germany) with reference to Fan’s [20] identification keys. 

#### 2.3.2. Molecular Identification

The COI common primers (LCO-1490 GGTCAACAAATCATAAAGATATTGG, HCO-2198 TAAACTTCAGGGTGACCAAAAAATCA) suggested by Folmer et al. [23] were employed for molecular identification of *C. nigripes*. Genomic DNA was extracted from the leg muscle tissue of adult samples using the Magen Hipure Insect DNA kit (MaBio Genomics, Inc., Gaithersburg, MD, USA), following the manufacturer’s instructions. The COI PCR amplification cycle parameters were set as follows: 94 °C for 2 min; 98 °C for 10 s, 51 °C for 15 s, 72 °C for 2.5 min, for a total of 35 cycles; and 72 °C for 10 min, then stored at 4 °C. After the PCR reaction, the products that matched the length of the target fragment were sent to Sangon Biotech Company (Shanghai, China) for sequencing. The resulting gene sequence was submitted to GenBank (accession number: OR044072).

### 2.4. Observation of the Egg Stage at Different Temperatures

Adults were reared at 28 °C (room temperature), and the eggs laid on the pig skin by the adults within 2 h were transferred to a new Petri dish with decomposed pork, moved to an insect-rearing box with 2 cm thick vermiculite at the bottom, and placed in intelligent light incubators. We found that the species occurred at 19–34 °C in field succession experiments as well as in actual cases. To obtain more comprehensive data, we conducted laboratory studies with the species at ±3 °C. We used eight intelligent light incubators set to 16 °C, 19 °C, 22 °C, 25 °C, 28 °C, 31 °C, 34 °C, and 37 °C, with humidity at 70%, and photoperiod L12:D12. A pre-experiment was performed to identify the time frame for hatching every hour. In subsequent formal experiments, to avoid repeated observations affecting the accuracy of the hatching time, observations were taken every half hour beginning two hours before the established hatching time window, and the hatching time was recorded; this process was repeated five times at each temperature.

### 2.5. Changes in Developmental Duration and Body Length at Different Temperatures

The larvae were reared in each of the eight intelligent light incubators after hatching, and the appropriate amount of decomposed pork was added to the Petri dishes daily based on their consumption. Starting from the 0th h of hatching, 6 larvae were randomly sampled every 4 h until pupariation, and the larvae were scalded in hot water above 90 °C and filled into vials (15 mL) containing 80% ethanol solution, which was subsequently used to identify the instar and measure the body length. Changes in posterior spiracle morphology were examined under a stereomicroscope to determine larval instar, and an electronic vernier caliper (Sangon, Shanghai, China) was used to measure larval body length with an accuracy of 0.01 mm. No further samples were collected after pupariation, and observations were made at 8 h intervals until eclosion, with the timings of different developmental events recorded. This experiment was repeated five times at each temperature.

### 2.6. Data Analysis

All statistical data analyses were conducted using Origin Pro 2019b, R4.3.0, and Python 3.7.0. A one-way ANOVA + LSD test was used to analyze the effect of temperature on the developmental period and T_L_ of different developmental events of *C. nigripes*. A scatter diagram was utilized to establish an isomorphen diagram based on the mean and standard deviation of the time required for seven developmental events at seven constant temperatures, with the time from oviposition (h) on the X-axis, temperature (°C) on the Y-axis. A contour plot was utilized to establish an isomegalen diagram, using the time after hatching (h) on the X-axis and the temperature (°C) on the Y-axis. The relationship between larval body length (mm) and time after hatching (h) was analyzed using a modified logistic equation to generate a function for estimating PMI_min_, as follows:

$$L\left(t\right)=\frac{L_{m}}{1+\left(\frac{L_{m}-L_{0}}{L_{0}}\right)e^{-\lambda t}}.$$

A linear regression model, as described by Ikemoto and Takai [24], was used to analyze the relationship between developmental time and accumulated degree hours (ADH) at each developmental stage, with the developmental time (h) on the x-axis and ADH on the y-axis. The slope of the resulting equation represents T_L_ and the intercept represents the thermal summation constant (K). A scatter diagram of each developmental event of *C. nigripes* was generated, with temperature (°C) on the x-axis and developmental rate (1/days) on the y-axis. T_L_, T_Φ_, and T_H_ were evaluated for each developmental stage of *C. nigripes* using the Ikemoto and Kiritani [25] proposed thermodynamic nonlinear model (Optim SSI). The expression for the SSI model is as follows:

$$\frac{\rho_{\emptyset }\frac{T}{T_{\emptyset }}\exp\left[\frac{∆H_{A}}{R}\left(\frac{1}{T_{\emptyset }}-\frac{1}{T}\right)\right]}{1+\exp\left[\frac{∆H_{L}}{R}\left(\frac{1}{T_{L}}-\frac{1}{T}\right)\right]+\exp\left[\frac{∆H_{H}}{R}\left(\frac{1}{T_{H}}-\frac{1}{T}\right)\right]}.$$

The R program developed by Ikemoto and Kiritani [25] was used to estimate the value of each parameter using R4.3.0 (https://www.r-project.org/ (accessed on 18 May 2023)).

## 3. Results

### 3.1. Developmental Duration and Isomorphen Diagram

*Chrysomya nigripes* could develop from egg to adult at a constant temperature range of 19–37 °C. However, in five replicate experiments, the developmental process was not completed at 16 °C, and the larvae started to die in large numbers in the middle and late second instar (about 316 h), with mortality rates as high as 90% and surviving individuals were late to enter the third instar and were small in size. Eventually, all larvae died and could not reach the intra-puparial period. As a result, no developmental data for *C. nigripes* were obtained at 16 °C. The overall developmental time consistently decreased as the temperature increased from 644.9 ± 36.8 h at 19 °C to 191.5 ± 3.8 h at 34 °C and then remained stable at 191.8 ± 2.0 h at 37 °C (Table 1), demonstrating that temperature significantly affected the developmental time of *C. nigripes*. Multiple comparisons between temperatures revealed that differences in the duration of the entire developmental process at 19 °C, 22 °C, 25 °C, and 28 °C were statistically significant (*p* < 0.05). In contrast, differences in the duration of the entire developmental process at 31 °C, 34 °C, and 37 °C were not statistically significant (*p* > 0.05). Therefore, the fastest developmental rate of *C. nigripes* should occur between 31 °C and 37 °C.

**Figure 1:**
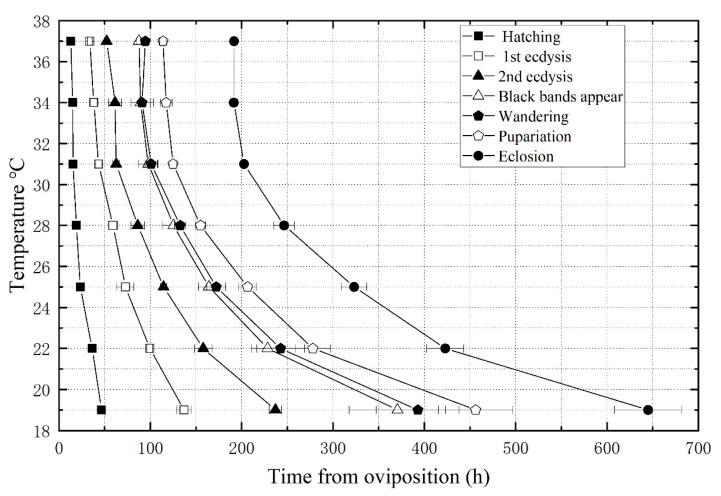
Isomorphen diagram of *Chrysomya nigripes*, showing the time (h) from oviposition to the onset of each developmental event (hatching, first instar, second instar, black bands appear, wandering, pupariation, and eclosion). Each line represents a developmental event, and the error bars are the standard deviation of the time each event occurred.

An isomorphen diagram was generated, showing different developmental events as different lines (Figure 1). Notably, the temperature and developmental time of *C. nigripes* showed a negative correlation in all seven developmental events. The larvae develop into the wandering stage soon after the black bands appear on their backs, so the lines representing the black bands’ appearance stage and the wandering stage in the isomorphen diagram are extremely close together.

### 3.2. Linear Thermal Summation Model and Optim SSI Model

**Figure 2:**
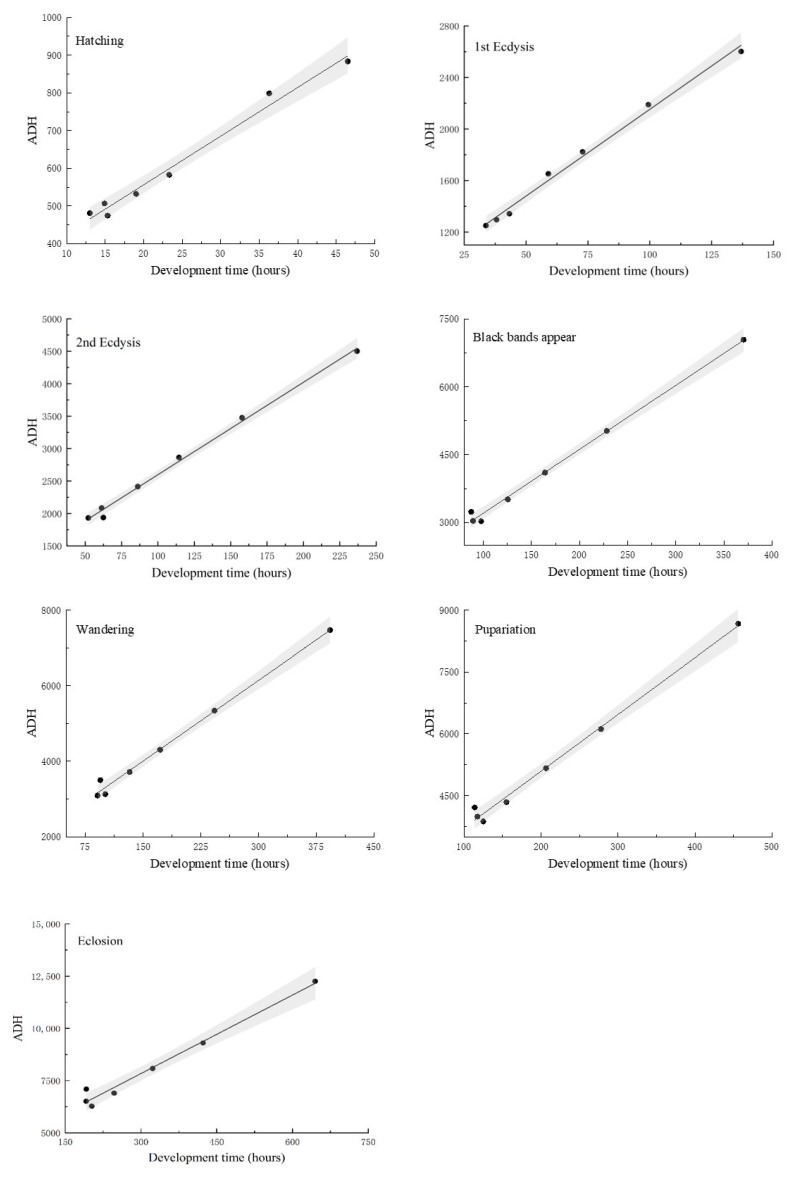
Linear thermal summation model of seven developmental events of *Chrysomya nigripes*. The area between the two dashed lines indicates the 95% confidence interval. The black dots denote the data used in the regression analysis, while the solid line denotes the regression line.

A linear thermal summation was established for the seven developmental events proposed by Ikemoto and Takai [24] (Figure 2). Furthermore, the relationship between the developmental time required by *C. nigripes* at different developmental stages, and ADH were linearly fitted to a straight line, and K and T_L_ corresponding to each developmental event were obtained using this model (Table 2). The regression coefficient R^2^ of each model was >0.97, demonstrating a strong linear association between developmental time and ADH. K and T_L_ of *C. nigripes* were 4083.00 ± 293.39 degree hour and 12.52 ± 0.83 °C, respectively, throughout the developmental period. T_L_ was above 14 °C for the second instar, black bands appear, and wandering stages, whereas it was lower for the hatching, first instar, pupariation, and eclosion stages.

**Figure 3:**
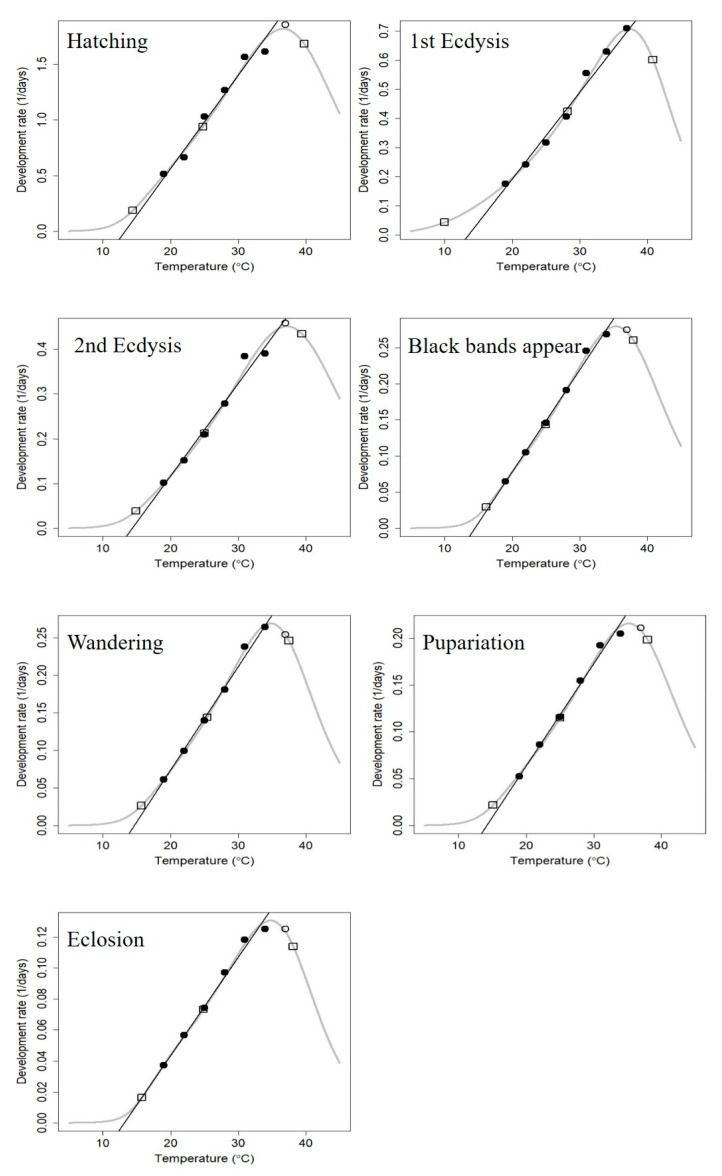
Nonlinear thermodynamic Optim SSI model for seven developmental events of *Chrysomya nigripes*. Circles indicate data points, and curves indicate the developmental rates predicted by the Optim SSI model. The three squares denote the predicted mean developmental rates of T_L_, T_Φ_, and T_H_. The black lines are obtained by linear fitting of the black circle data, whereas the white circles are the data excluded from the linear fitting.

The nonlinear thermodynamic Optim SSI model proposed by Ikemoto and Kiritani [25] was used to fit the seven developmental events of *C. nigripes* at 19–37 °C (Figure 3). The scatter diagram demonstrated that the highest temperature data point in this study, 37 °C, was not linearly related to the temperature of the remaining six events, except for the first instar developmental stage, which was part of a nonlinear curve model. The results showed that T_L_, T_Φ_, and T_H_ of *C. nigripes* were 15.76 °C, 24.88 °C, and 38.15 °C, respectively (Table 3), and the Optim SSI model was up-regulated compared with the linear model for T_L_ except for the first instar, and T_L_ reached 16.15 °C for the black bands appear stage, which explained the phenomenon that *C. nigripes* died a lot at 16 °C before entering the black bands appear stage at the end of the third instar.

### 3.3. Larval Body Length Variation and Isomegalen Diagram 

**Figure 4:**
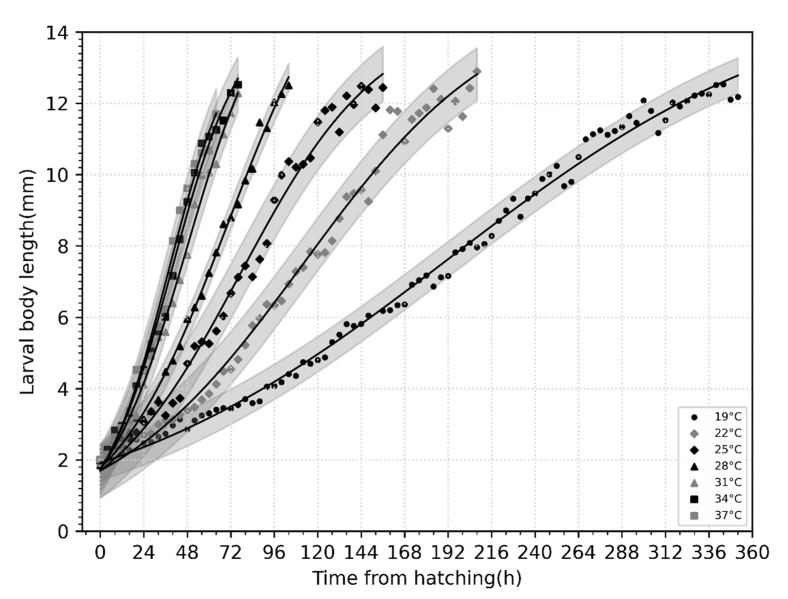
Variation of body length with time (h) in larvae of *Chrysomya nigripes* at different constant temperatures.

A plot of variation in body length with time (h) in larvae of *C. nigripes* at different constant temperatures (Figure 4) demonstrated that the body length of newly hatched larvae was approximately 2 mm, and they developed rapidly until the wandering stage when their body length was between 12–13 mm. The relationship between the age and length of the larvae was described using a general model represented by a logistic equation (Table 4).

**Figure 5:**
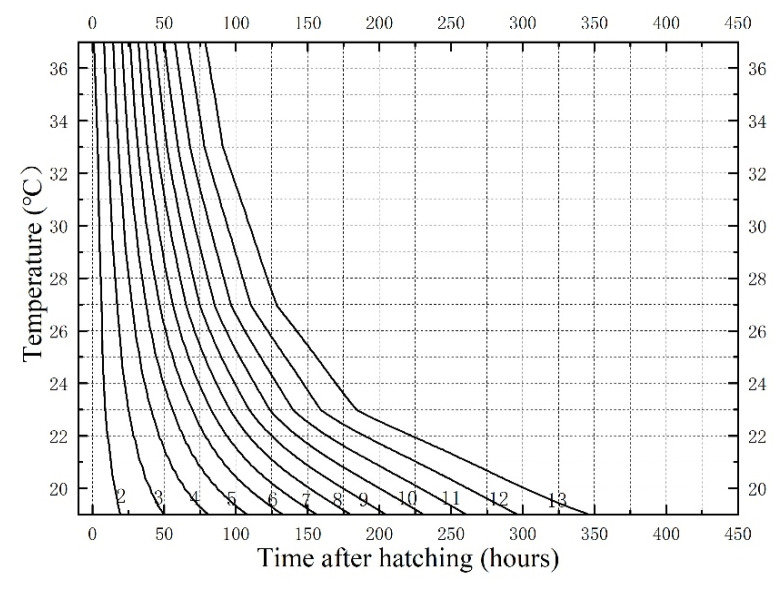
Isomegalen diagram of *Chrysomya nigripes*. Time (h) is plotted against temperature (°C), and each line represents the length (mm) of the developing larvae (2–13). The number at the bottom right of each fold denotes the length.

In the isomegalen diagram (Figure 5), the number on each contour (2–13) corresponds to a larval body length, the coordinates of the X-axis representing the time required to develop to that body length at the temperature corresponding to the Y-axis—the time after hatching served as the starting point.

### 3.4. The Application of C. nigripes in PMI_min_ Estimation 

**Figure 6:**
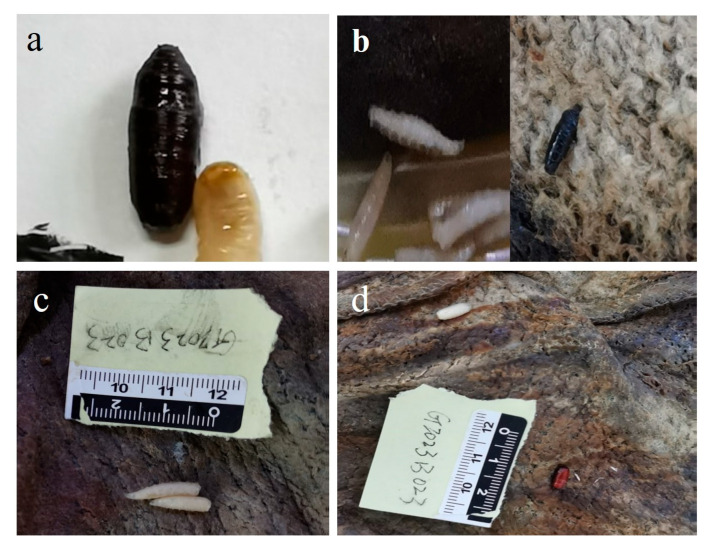
Insect evidence found at the death scene: (**a**) *Chrysomya megacephala* pupae; (**b**) *Chrysomya nigripes* larvae (left) and *Chrysomya nigripes* pupae (right); (**c**) *Muscina stabulans* larvae; (**d**) *Muscina stabulans* pupae (lower right).

A corpse was discovered on the bed in a first-floor residential room in Zhongshan City, Guangdong Province, China, on 30 March 2023, at 23:30 p.m., with the bedroom window closed and the door open. The doors and windows of other rooms were closed, and there was no central temperature control system. The corpse in a state of advanced decomposition was lying on its back, with the upper half of the body lying on the bed and the feet hanging on the side of the bed; the lower abdomen, right lower limb, and right thigh of the corpse were covered with a quilt. The insect evidence found on the corpse was mostly distributed in the area covered with a quilt. The corpse was returned to the autopsy room of the Public Security Bureau for postmortem examination and collection of insect evidence on March 31. The pupae of *Chrysomya megacephala* (Fabricius, 1794) (Figure 6a), the larvae and pupae of *C. nigripes* (Figure 6b), and the larvae and pupae of *Muscina stabulans* (Fallén 1817) (Figure 6c,d) were found on the corpse. The average temperature of the 12 days before the corpse was found was 21.82 °C, according to the data from the nearest meteorological station to the site. It was estimated that it takes 200 h for *C. megacephala* [26] and 254 h for *M. stabulans* [27] to pupariate at this temperature. Based on the data in Figure 1 of this work, it was estimated that it takes 284 h for *C. nigripes* to pupariate at this temperature. In this view, we could estimate that the PMI_min_ of the corpse should be no less than 284 h (11.8 days). The deceased was last seen by the family 12 days ago, according to the police inquiry, and the water meter at the deceased’s residence likewise revealed no traces of water consumption since that time; this is consistent with the time of death we estimated using the developmental data of *C. nigripes*.

## 4. Discussion

*Chrysomya nigripes* is a species of the Calliphoridae family; however, unlike other species, *C. nigripes* appears later and stays on corpses for a longer time. In Guam, Bohart and Gressitt [28] discovered that the larvae of this species are a late secondary colonizer of carrion, including human corpses. O’Flynn and Moorhouse [29] found that *C. nigripes* prefers highly decaying carrion over *Chrysomya rufifacies* (Macquart, 1842), *Chrysomya varipes* (Macquart, 1850), and *Chrysomya saffranea* (Bigot, 1877). Because, unlike the three species, *C. nigripes* was not detected at an early stage of body decomposition, this is consistent with our findings when we collected this species: when *C. nigripes* appeared, the carcass was already highly decomposed. However, in our case report, PMI_min_ was comparable to the true PMI. In succession experiments by Ahmad and Ahamd [30], *C. nigripes* arrived at the carcass on the first day during the dry or rainy season. The case reported in this study also occurred during a rainy season, and the fly may have colonized the corpse shortly after death. This phenomenon may be best explained by the effect of the quilt on the growth and development of sarcosaprophagous insects. Larvae and pupae were largely distributed in the area covered by the quilt since the body was discovered covered in it. As a result, the heat generated during body decomposition could not be lost due to the covering of the quilt, and the larval mass and microbial activities also generated heat, resulting in a higher temperature in the area covered by the quilt. In this view, *C. nigripes* pupariated in a shorter time, compensating for the pre-colonization time of *C. nigripes*.

**Figure 7:**
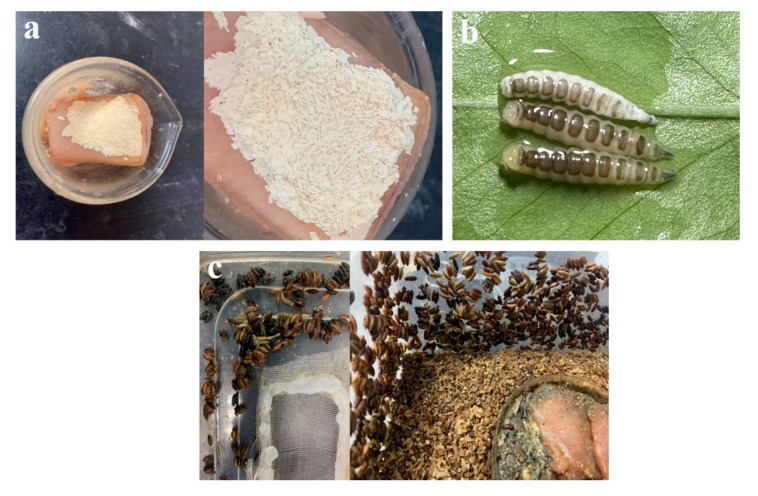
Special biological characteristics of *Chrysomya nigripes*: (**a**) sheet-like egg mass with moldy appearance; (**b**) black bands in each dorsal segment of the third instar larvae; (**c**) pupae attached to the lid and walls of the box.

Most research on *C. nigripes* currently focuses on morphology ultrastructure and molecular aspects [31,32,33,34]; however, there are limited data on the biological characteristics of this species. Our study revealed that *C. nigripes* has unique behavioral characteristics. Unlike other Calliphoridae species, which lay their eggs in the mouth, nose, and anus, *C. nigripes* adults lay their eggs directly on the skin surface of the carcasses. Unlike the clumpy egg mass laid by other blow flies, the egg mass is sheet-like, like a layer of mold (Figure 7a). These flaky egg clusters are structured so that predators, such as ants, find them difficult to bring back to the nest, and the risk of predation is significantly lower than that of clumpy eggs. According to the field experiment findings, we covered the decomposed pork with a layer of pig skin to induce the oviposition of *C. nigripes* in the laboratory. *Chrysomya nigripes* will lay a high number of eggs on the surface of the pig skin, but the number of eggs is relatively low when only decomposed pork is used to induce eggs. Secondly, *C. nigripes* produces black bands on its back in the late third instar (Figure 7b). Our observation revealed that *C. nigripes* enters the wandering stage shortly after producing black bands, beginning to crawl slowly or stop moving to prepare for pupariation, and the black bands persist. The black bands on the back of the larvae give *C. nigripes* the appearance of aggressive beetle larvae, making them safer in a potentially dangerous cadaver environment. In addition, unlike other Calliphoridae species, the larvae do not pupariate in the soil around the body but pupariate on the surface of the body or soil following the wandering period. Some individuals ascend higher to the vegetation or walls around the body to pupariate. This pupariation behavior aids investigators at the death scene in locating the species’ pupae or puparium. The larvae pupariate on the walls and lid of the rearing box while being reared in the laboratory (Figure 7c); this is consistent with our field experiment results. Before pupariating, the larvae secrete mucus that maintains the puparium tightly attached to the object, preventing it from being blown away by the wind or removed by predators, including ants and wasps. The biological characteristics of *C. nigripes* listed above are self-defense strategies developed in the long-term evolutionary process to defend themselves from predators or from being destroyed by the natural environment. The molecular mechanism behind these intriguing biological traits deserves additional investigation. 

Developmental data on *C. nigripes* have only been reported by O’Flynn [21] and Li et al. [22]. O’Flynn only provided limited data on developmental duration at 28 °C, with no available data for the egg stage, a total of 2–5 days for the first and second instars, 5–10 days for the entire larval stage, and 4–5 days for the intra-puparial period. Our findings reveal 2.8 days for the first and second instars, 5.7 days for the complete larval stage, and 3.8 days for the intra-puparial period at 28 °C, which are within the range of O’Flynn. Li studied 6 developmental events of *C. nigripes* at four temperatures, 20 °C, 24 °C, 28 °C and 32 °C, sampled the larvae at 12 h intervals, 3 replicates were set up, and scatter diagrams were plotted for the variation of larval body length with time, and the mean developmental duration of *C. nigripes* at 20 °C, 24 °C, 28 °C, and 32 °C was 608 h, 327 h, 254 h, and 217 h, respectively. Our data essentially matched with their developmental data at each temperature, and we showed that the mean developmental duration of *C. nigripes* at 19 °C, 25 °C, 28 °C, and 31 °C were 644.9 h, 323.1 h, 246.6 h, and 202.5 h, respectively, validating the accuracy of the results of this study.

Our study included new developmental data and multiple data models compared to earlier research. First, we studied seven developmental events at seven different temperatures (19 °C, 22 °C, 25 °C, 28 °C, 31 °C, 34 °C, and 37 °C), with samples every 4 h. We explored a wider temperature range with shorter sampling intervals, obtaining more accurate results. Furthermore, we treated the appearance of the black bands as a separate developmental event and discovered that it was closely related to the wandering event. Second, we found that the intra-puparial period of *C. nigripes* is short, accounting for less than 40% of the whole developmental duration, in contrast to other species in the genus *Chrysomya*, where the intra-puparial period of *C. megacephala* [35] and *C. rufifacies* [36] can reach 50–70% of the whole developmental duration. Finally, we estimated T_L_ for *C. nigripes* using the linear thermal summation model and the nonlinear thermodynamic Optim SSI model. The linear thermal summation model demonstrated that T_L_ for the second instar, black bands appear, and wandering stages were about 14 °C, whereas the Optim SSI model calculated a higher value of T_L_, reaching 16 °C. These results were consistent with the fact that *C. nigripes* larvae developed slowly and died in large numbers in the late second instar at 16 °C in this experiment, and, in agreement with Li’s [22] results, he also did not obtain developmental data for *C. nigripes* at 16 °C. Marchenko [37] revealed T_L_ for several species of blow flies as follows: *Calliphora vicina* Robineau-Desvoidy, 1830, 2.0 °C; *Calliphora vomitoria* (Linnaeus, 1758), 3.0 °C; *Protophormia terraenovae* (Robineau-Desvoidy, 1830), 7.8 °C; *Lucilia sericata* Meigen, 1826, 9.0 °C; *Chrysomya albiceps* (Wiedemann, 1819), 10.2 °C; *Phormia regina* (Meigen, 1826), 11.4 °C. In view of these findings, the T_L_ of *C. nigripes* was higher in comparison to the other blow fly species.

There have been few studies on the high threshold temperature of blow flies. Our experimental results show that the developmental duration of *C. nigripes* is longer at 37 °C than at 34 °C, indicating that this temperature is close to the maximum tolerance temperature of this species. These data are comparable to T_H_ of 38.15 °C calculated using the SSI model, which is also consistent with the distribution of *C. nigripes* in tropical and subtropical regions and southern China. In contrast to T_H_ of *C. nigripes*, some fly species, such as *M. stabulans* [27] and *Megaselia scalaris* [38] in the same region, do not exceed 34 °C for the upper critical thermal threshold.

## 5. Conclusions

The present study set a more comprehensive temperature range, covering the minimum to maximum temperatures at which individuals can develop in their natural environment. Furthermore, we used several analytical approaches to construct several data models and incorporated the logistic equation and an improved nonlinear thermodynamic Optim SSI model to obtain more reliable and robust data. This study focuses on the morphology of the species to obtain data that can be used to estimate PMI_min_; in addition, PMI_min_ can be estimated more accurately when combined methodologically with spectroscopy and omics, and disciplinarily with forensic anthropology related to total body score (TBS)/accumulated degree days (ADDs), and forensic pathology decomposition stages.

## Figures and Tables

**Table 1:** Developmental duration (mean ± standard deviation, h) of seven developmental events at seven constant temperatures, 70% humidity, and a photoperiod of 12:12 h L:D.

Developmental Events	Temperature (°C)						
	19 °C	22 °C	25 °C	28 °C	31 °C	34 °C	37 °C
Hatching	46.5 ± 2.4	36.3 ± 0.4	23.3 ± 0.2	19.0 ± 0.8	15.3 ± 1.2	14.9 ± 0.7	13.0 ± 1.2
First ecdysis	136.9 ± 8.0	99.5 ± 3.3	72.9 ± 9.2	59.0 ± 4.8	43.3 ± 3.2	38.1 ± 3.8	33.8 ± 2.6
Second ecdysis	236.9 ± 6.7	157.9 ± 9.5	114.5 ± 3.5	86.2 ± 7.3	62.5 ± 3.3	61.3 ± 7.1	52.2 ± 2.6
Black bands appear	370.5 ± 52.7	228.3 ± 17.6	164.1 ± 11.2	125.4 ± 11.5	97.7 ± 10.6	89.3 ± 10.6	87.4 ± 2.5
Wandering	392.9 ± 45.4	242.7 ± 25.9	172.1 ± 10.3	132.6 ± 6.0	100.9 ± 6.9	90.9 ± 12.3	94.6 ± 2.2
Pupariation	456.1 ± 40.8	277.9 ± 19.0	206.5 ± 9.7	155.0 ± 5.6	124.9 ± 3.9	117.3 ± 6.5	113.8 ± 3.4
Eclosion	644.9 ± 36.8 ^a^	422.9 ± 20.1 ^b^	323.1 ± 13.9 ^c^	246.6 ± 11.2 ^d^	202.5 ± 1.8 ^e^	191.5 ± 3.8 ^e^	191.8 ± 2.0 ^e^

In one-way ANOVA + LSD test, values with the same letter are not statistically different at *p* > 0.05.

**Table 2:** Mean values (±SE) of the lower critical thermal threshold (T_L_) and thermal summation constant (K) for the seven developmental events of *Chrysomya nigripes* and the coefficient of determination (R^2^) of the linear thermal summation model.

Developmental Stage	K ± SE (Degree Hours)	T_L_ ± SE (°C)	R^2^
Hatching	298.02 ± 19.58	12.91 ± 0.73 ^ab^	0.98
First ecdysis	806.38 ± 39.21	13.46 ± 0.51 ^bc^	0.99
First ecdysis	1174.29 ± 55.43	14.24 ± 0.44 ^c^	0.99
Black bands appear	1792.52 ± 87.52	14.13 ± 0.46 ^c^	0.99
Wandering	1868.06 ± 116.42	14.24 ± 0.57 ^c^	0.99
Pupariation	2340.76 ± 135.44	13.76 ± 0.57 ^c^	0.99
Eclosion	4083.00 ± 293.39	12.52 ± 0.83 ^a^	0.97

In one-way ANOVA + LSD test, values with the same letter are not statistically different at *p* > 0.05.

**Table 3:** Parameters of the different developmental events of *Chrysomya nigripes* estimated using the nonlinear thermodynamic Optim SSI model.

Parameter (Unit)	Hatching	First Ecdysis	First Ecdysis	Black Bands Appear	Wandering	Pupariation	Eclosion
T_Φ_ (°C)	24.78	28.17	25.03	24.93	25.40	25.04	24.88
ρ_Φ_ (day^−1^)	0.97	0.44	0.22	0.15	0.15	0.12	0.75
∆H_A_ (cal/mol)	1.48 × 10^4^	1.45 × 10^4^	1.70 × 10^4^	1.72 × 10^4^	1.77 × 10^4^	1.66 × 10^4^	1.49 × 10^4^
∆H_L_ (cal/mol)	−7.30 × 10^4^	−3.69 × 10^4^	−6.95 × 10^4^	−8.63 × 10^4^	−7.55 × 10^4^	−7.37 × 10^4^	−9.29 × 10^4^
∆H_H_ (cal/mol)	5.03 × 10^4^	6.77 × 10^4^	4.85 × 10^4^	5.67 × 10^4^	6.25 × 10^4^	5.75 × 10^4^	6.34 × 10^4^
T_L_ (°C)	14.42	9.98	14.89	16.15	15.67	15.09	15.76
T_H_ (°C)	39.75	40.85	39.42	37.93	37.50	38.00	38.15
χ^2^	2.43 × 10^−2^	1.89 × 10^−3^	4.81 × 10^−3^	3.51 × 10^−4^	2.29 × 10^−4^	8.81 × 10^−4^	3.32 × 10^−4^
R^2^	0.980	0.996	0.983	0.998	0.999	0.994	0.995

**Table 4:** A general model describing the relationship between the body length of *Chrysomya nigripe* larvae (L) (mm) and the time after hatching (t) (hour).

Model	
$L\left(t\right)=\frac{L_{m}}{1+\left(\frac{L_{m}-L_{0}}{L_{0}}\right)e^{-\lambda t}}$	(1)
$L_{0}\left(T\right)=1.9014\;-0.0041T$	(2a)
$L_{m}\left(T\right)=15.2812\;-\;0.0310T$	(2b)
$\left(T\right)=-0.0396+0.0027T$	(2c)

L(t) is the larval length at time t. L_0_ is the initial length (i.e., at t = 0), L_m_ is the final length, and λ is a parameter expressing growth rate. Equation (2a–c) model temperature (T) effects on L_0_, L_m_, and λ.

## Data Availability

The data presented in this study are available in Supplementary Materials.

## Supplementary Materials

The following supporting information can be downloaded at: https://www.mdpi.com/article/10.3390/insects14090729/s1, Table S1: larval body length data of *C. nigripes*. Table S2: developmental duration data of *C. nigripes.*

## References

[1] Lord W., Adkins T., Catts E. (1992). The use of *Synthesiomyia nudesita* (Van Der Wulp) (Diptera: Muscidae) and *Calliphora vicina* (Robineau-Desvoidy) (Diptera: Calliphoridae) to estimate the time of death of a body buried under a house. J. Agric. Entomol..

[2] Smith K.G.V. (1986). A Manual of Forensic Entomology.

[3] Hu C., Min J., Wang J. (2000). Forensic Entomology.

[4] HB J., LC J. (2001). Forensic Entomology: The Utility of Arthropods in Legal Investigations.

[5] Zhao H., Liu C. (2021). Advanced Forensic Medicine.

[6] Wang J. (2019). Practical Forensic Entomology.

[7] Vanin S., Gherardi M., Bugelli V., Di Paolo M. (2011). Insects found on a human cadaver in central Italy including the blowfly *Calliphora loewi* (Diptera, Calliphoridae), a new species of forensic interest. Forensic Sci. Int..

[8] Dekeirsschieter J., Frederickx C., Verheggen F.J., Boxho P., Haubruge E. (2013). Forensic entomology investigations from Doctor Marcel Leclercq (1924–2008): A review of cases from 1969 to 2005. J. Med. Entomol..

[9] Syamsa R., Omar B., Zuha R., Faridah M., Hidayatulfathi O., Shahrom A. (2015). Forensic entomology of high-rise buildings in Malaysia: Three case reports. Trop. Biomed..

[10] Lutz L., Zehner R., Verhoff M.A., Bratzke H., Amendt J. (2021). It is all about the insects: A retrospective on 20 years of forensic entomology highlights the importance of insects in legal investigations. Int. J. Legal. Med..

[11] Tarone A.M., Sanford M.R. (2017). Is PMI the Hypothesis or the Null Hypothesis?. J. Med. Entomol..

[12] Byrd J.H.U.O., Butler J.F. (1996). Effects of temperature on *Cochliomyia macellaria* (Diptera: Calliphoridae) development. J. Med. Entomol..

[13] Byrd J.H., Butler J.F. (1998). Effects of temperature on *Sarcophaga haemorrhoidalis* (Diptera: Sarcophagidae) development. J. Med. Entomol..

[14] Campobasso C.P., Di Vella G., Introna F. (2001). Factors affecting decomposition and Diptera colonization. Forensic Sci. Int..

[15] Lord W., Goff M., Adkins T., Haskell N. (1994). The black soldier fly *Hermetia illucens* (Diptera: Stratiomyidae) as a potential measure of human postmortem interval: Observations and case histories. J. Forensic Sci..

[16] Sukontason K., Narongchai P., Kanchai C., Vichairat K., Sribanditmongkol P., Bhoopat T., Kurahashi H., Chockjamsai M., Piangjai S., Bunchu N. (2007). Forensic entomology cases in Thailand: A review of cases from 2000 to 2006. Parasitol. Res..

[17] Silahuddin S.A., Latif B., Kurahashi H., Walter D.E., Heo C.C. (2015). The importance of habitat in the ecology of decomposition on rabbit carcasses in Malaysia: Implications in forensic entomology. J. Med. Entomol..

[18] Syamsa R.A., Omar B., Ahmad F.M.S., Hidayatulfathi O., Shahrom A.W. (2017). Comparative fly species composition on indoor and outdoor forensic cases in Malaysia. J. Forensic Leg. Med..

[19] Sukontason K.L., Kanchai C., Piangjai S., Boonsriwong W., Bunchu N., Sripakdee D., Chaiwong T., Kuntalue B., Siriwattanarungsee S., Sukontason K. (2006). Morphological observation of puparia of *Chrysomya nigripes* (Diptera: Calliphoridae) from human corpse. Forensic Sci. Int..

[20] Fan Z. (1992). The Identification Keys of Common Flies in China.

[21] O’Flynn M.A. (1983). The succession and rate of development of blowflies in carrion in Southern Queensland and the application of these data to forensic entomology. Aust. J. Entomol..

[22] Li L., Wang Y., Wang J., Ma M., Lai Y. (2016). Temperature-dependent development and the significance for estimating postmortem interval of *Chrysomya nigripes* Aubertin, a new forensically important species in China. Int. J. Legal. Med..

[23] Folmer O., Black M., Hoeh W., Lutz R. (1994). DNA primers for amplification of mitochondrial cytochrome c oxidase subunit I from diverse metazoan invertebrates. Mol. Mar. Biol. Biotechnol..

[24] Ikemoto T., Takai K. (2000). A new linearized formula for the law of total effective temperature and the evaluation of line-fitting methods with both variables subject to error. Environ. Entomol..

[25] Ikemoto T., Kiritani K. (2019). Novel method of specifying low and high threshold temperatures using thermodynamic SSI model of insect development. Environ. Entomol..

[26] Zhang Y., Wang Y., Yang L., Tao L., Wang J. (2018). Development of *Chrysomya megacephala* at constant temperatures within its colony range in Yangtze River Delta region of China. Forensic Sci. Res..

[27] Wang Y., Hu G., Zhang Y., Wang M., Amendt J., Wang J. (2019). Development of *Muscina stabulans* at constant temperatures with implications for minimum postmortem interval estimation. Forensic Sci. Int..

[28] O’Flynn M.A. (1983). Notes on the Biology of *Chrysomya nigripes* Aubertin (Diptera: Calliphoridae). Aust. J. Entomol..

[29] O’Flynn M., Moorhouse D.E. (1979). Species of *Chrysomya* as primary flies in carrion. Aust. J. Entomol..

[30] Ahmad A., Ahmad A.H. (2009). A preliminary study on the decomposition and dipteran associated with exposed carcasses in an oil palm plantation in Bandar Baharu, Kedah, Malaysia. Trop. Biomed..

[31] Sukontason K.L., Vogtsberger R.C., Boonchu N., Chaiwong T., Sripakdee D., Ngern-klun R., Piangjai S., Sukontason K. (2005). Larval morphology of *Chrysomya nigripes* (Diptera: Calliphoridae), a fly species of forensic importance. J. Med. Entomol..

[32] Sukontason K.L., Chaiwong T., Piangjai S., Upakut S., Moophayak K., Sukontason K. (2008). Ommatidia of blow fly, house fly, and flesh fly: Implication of their vision efficiency. Parasitol. Res..

[33] Szpila K., Wallman J.F. (2016). Morphology and identification of first instar larvae of Australian blowflies of the genus *Chrysomya* of forensic importance. Acta Trop..

[34] Zhang Z.Y., Ren L.P., Leng J., Shang Y.J., Zhu G.H. (2019). The complete mitochondrial genome of *Chrysomya nigripes* (Diptera: Calliphoridae). Mitochondrial DNAB.

[35] Gruner S.V., Slone D.H., Capinera J.L., Turco M.P. (2017). Development of the Oriental latrine Fly, *Chrysomya megacephala* (Diptera: Calliphoridae), at five constant temperatures. J. Med. Entomol..

[36] Yanmanee S., Husemann M., Benbow M.E., Suwannapong G. (2016). Larval development rates of *Chrysomya rufifacies* Macquart, 1842 (Diptera: Calliphoridae) within its native range in South-East Asia. Forensic Sci. Int..

[37] Marchenko M.I. (2001). Medicolegal relevance of cadaver entomofauna for the determination of the time of death. Forensic Sci. Int..

[38] Zhang Y., Li L., Liao M., Kang C., Hu G., Guo Y., Wang Y., Wang J. (2023). Development of *Megaselia scalaris* at constant temperatures and its significance in estimating the time of death. Int. J. Leg. Med..

